# Research on the circadian clock gene HNF4a in different malignant tumors

**DOI:** 10.7150/ijms.49997

**Published:** 2021-01-21

**Authors:** Meng-jun Qiu, Li Zhang, Xie-fan Fang, Qiu-ting Li, Li-sheng Zhu, Bin Zhang, Sheng-li Yang, Zhi-fan Xiong

**Affiliations:** 1 Division of Gastroenterology, Liyuan Hospital, Tongji Medical College, Huazhong University of Science and Technology, Wuhan 430077, China.; 2 Charles River Laboratories, Inc., 6995 Longley Lane, Reno NV 89511.; 3 Cancer Center, Union Hospital, Tongji Medical College, Huazhong University of Science and Technology, Wuhan 430022, China.

**Keywords:** circadian clock, HNF4a, malignant tumors, prognosis.

## Abstract

**Background:** The circadian rhythm is produced by multiple feedback loops formed by the core clock genes after transcription and translation, thus regulating various metabolic and physiological functions of the human body. We have shown previously that the abnormal expression of 14 clock genes is related closely to the occurrence and development of different malignant tumors, and these genes may play an anti-cancer or pro-cancer role in different tumors. HNF4a has many typical properties of clock proteins involved in the clock gene negative feedback loop regulation process. We need to explore the function of HNF4a as a circadian clock gene in malignant tumors further. **Methods:** We used The Cancer Genome Atlas (TCGA) database to download the clinicopathological information of twenty malignant tumors and the corresponding RNA-seq data. The HNF4a RNA-seq data standardized by R language and clinical information were integrated to reveal the relationship between HNF4a and prognosis of patients. **Results:** Analysis of TCGA data showed that the prognosis of HNF4a was significantly different in BLCA, KIRC, LUSC, and READ. High HNF4a expression is correlated with good prognosis in BLCA, KIRC, and READ but poor prognosis in LUSC. However, HNF4a was associated with the stages, T stages, and lymph node status only in BLCA. **Conclusions:** HNF4a plays different roles in different malignancies, and the abnormal expression of HNF4a has a great correlation with the biological characteristics of BLCA. The low expression of HNF4a could be a reference index for the metastasis, recurrence, and prognosis of BLCA.

## Introduction

HNF4a is a member of the nuclear receptor superfamily HNF4, and is also a hepatic cell-specific transcription factor. In addition to its expression in the liver, HNF4a is commonly expressed in organs containing epithelial cells, whose transcriptional translation involves a regulated expression of many activity-related target genes, such as lipid transport, glucose, cholesterol, amino acid metabolism, and the regulation of the blood system 1-4. Changes in physiological activity, such as changes in insulin levels after a meal and changes in the products of dietary metabolisms, such as bile acids, can affect the expression level of HNF4a 5-7. The importance of maintaining physiological homeostasis is reflected in the function of HNF4a in drug metabolism and its potential as a drug target 8.

The poor differentiation and immortalization of cells are the bases of tumorigenesis. It has been shown that HNF4a can not only reduce or inhibit cell proliferation but can also accelerate the differentiation speed of poorly differentiated liver cancer by re-expressing HNF4a in liver cancer and maintaining a highly differentiated phenotype, which reduces its invasiveness 9-11. Cell growth includes proliferation and apoptosis, and the comprehensive effects of the two are ultimately manifested as the strength of cell growth ability. A cell cycle closely related to apoptosis is also an important factor in tumor development, and the overexpression of HNF4a has been found to inhibit G1 cell cycle progression in reported work 12.

Circadian rhythm is a physiological, biochemical, and behavioral activity that organisms used to adapt to periodic changes in the environment 13. The basic working principle of this activity relies mainly on the regulation of the transcription of the core clock element to achieve the consistency of the rhythm of each organ of the body with the circadian rhythm of the external environment 14. At the molecular level, maintaining the normal function of the circadian rhythm involves the negative feedback loop formed by a series of clock genes, namely the transcription/translation negative feedback, which regulates circadian rhythms at the cellular level, governing sleep, eating, temperature, hormonal secretion, and a number of metabolic pathways 14, 15. The disruption of the normal circadian rhythm can lead to clinically relevant diseases, including cardiovascular diseases, neurodegenerative diseases, diabetes, and various cancers 16-20.

HNF4a is a clock gene that is mainly studied in the field of liver cancer. It can play an anti-cancer role by inhibiting the initiation, growth of hepatocellular carcinoma, or progressions of some genes, such as cyclin D1 and CCND1 9, 21-23, and act as a transcriptional activator, interacting with repressor proteins to allow the inhibition of some target genes 24-26. The gene can also affect apoptosis and cell cycle by upregulating Exo70 and inhibiting the epithelial-mesenchymal transformation gene or inhibit the expression of the circadian protein BMAL1 directly 27, 28. The exact association between HNF4a and cancers remains unclear. In this work, the TCGA database was used to analyze the expression of HNF4a in twenty malignant tumors (adrenocortical carcinoma (ACC), bladder urothelial carcinoma (BLCA), breast invasive carcinoma (BRCA), cervical squamous cell carcinoma and endocervical adenocarcinoma (CESC), cholangiocarcinoma (CHOL), colon adenocarcinoma (COAD), lymphoid neoplasm diffuse large B-cell Lymphoma (DLBC), esophageal carcinoma (ESCA), head and neck squamous cell carcinoma (HNSC), kidney renal clear cell carcinoma (KIRC), kidney renal papillary cell carcinoma (KIRP), liver hepatocellular carcinoma (LIHC), lung adenocarcinoma (LUAD), lung squamous cell carcinoma (LUSC), ovarian serous cystadenocarcinoma (OV), pancreatic adenocarcinoma (PAAD), rectum adenocarcinoma (READ), sarcoma (SARC), stomach adenocarcinoma (STAD), uterine corpus endometrial carcinoma (UCEC)) to evaluate the patient prognoses and clinicopathological features.

## Materials and methods

The RNA-Seq of HNF4a in twenty different malignant tumors and the corresponding clinical data were downloaded from the TCGA website (https://cancergenome.nih.gov/). The RNA-Seq data from 79 ACC tissues, 408 BLCA tissues, 1093 BRCA tissues, 304 CESC tissues, 36 CHOL tissues, 457 COAD tissues, 48 DLBC tissues, 184 ESCA tissues, 520 HNSC tissues, 533 KIRC tissues, 290 KIRP tissues, 371 LIHC tissues, 515 LUAD tissues, 501 LUSC tissues, 304 OV tissues, 178 PAAD tissues, 105 READ tissues, 259 SARC tissues, 415 STAD tissues, and 545 UCEC tissues. In this study, we integrated the RNA-Seq data standardized by the software edgeR from Bioconductor (http://www.bioconductor.org/) and clinical data according to the serial number of each patient and specimen provided by TCGA. Since the above two types of data are not exactly matched, we performed the culling of the mismatched data. Finally, the analysis of the prognoses and clinicopathological data of HNF4a in twenty different malignant tumors was performed on the data from 79 ACC tissues, 405 BLCA tissues, 1092 BRCA tissues, 304 CESC tissues, 36 CHOL tissues, 298 COAD tissues, 48 DLBC tissues, 184 ESCA tissues, 519 HNSC tissues, 533 KIRC tissues, 289 KIRP tissues, 370 LIHC tissues, 507 LUAD tissues, 495 LUSC tissues, 303 OV tissues, 178 PAAD tissues, 97 READ tissues, 259 SARC tissues, 408 STAD tissues, and 181 UCEC tissues. We assessed the relationship between the differential expression of HNF4a according to the median of the data and survival time and finally made the survival curve. We also examined the correlation between HNF4a expression and associated clinicopathological information with a variable degree of deletion in those cancers which has significantly different prognosis. Finally, we used the Genotype-Tissue Expressions (GTEx) database (http://commonfund.nih.gov/GTEx/) to analyze the expression differences of HNF4a in various normal tissues. Statistical analysis was performed with the SPSS® version 18.0. The variance analysis of the effects of HNF4a on prognosis was performed using Kaplan-Meier survival curves and the log-rank test. The indexes between the HNF4a expression and clinicopathological information, as well as the correlation between HNF4a and other clock genes, were compared using Pearson's Chi-square test.

## Results

**Figure 1:**
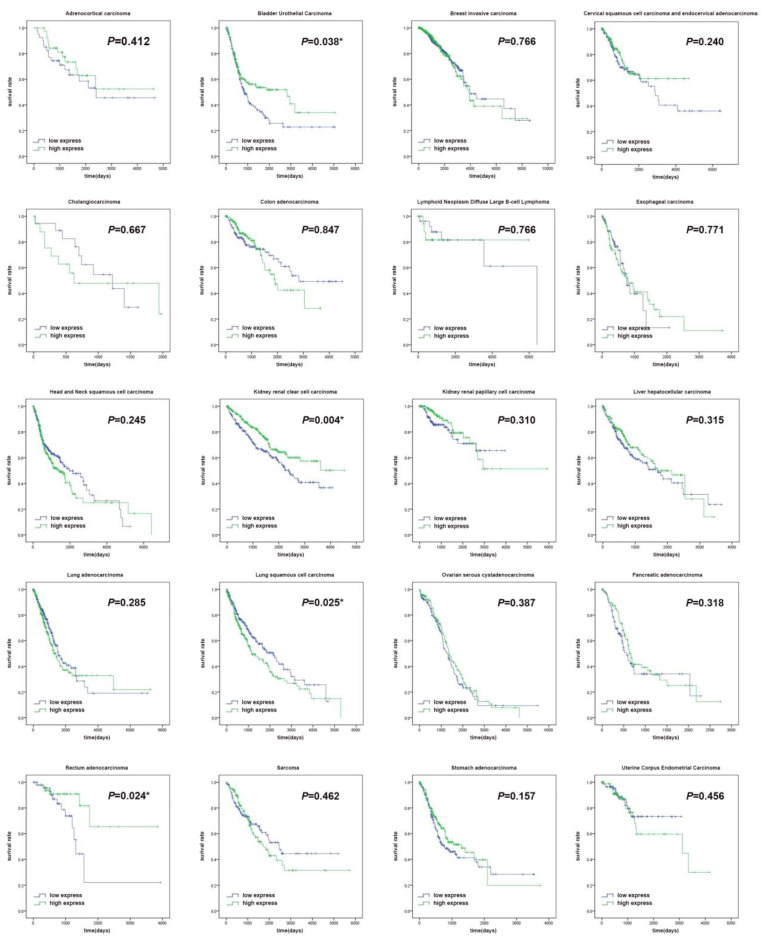
Survival curves of the clock gene HNF4a in twenty malignant tumors. The survival curves showed that patients with high expression of HNF4a exhibited a significantly longer overall survival (OS, *p* < 0.05) than patients with low HNF4a expression in BLCA, KIRC, and READ, while patients with high expression of HNF4a exhibited a significantly shorter OS (*p* < 0.05) than patients with low HNF4a expression in LUSC. No significant difference was observed in other malignant tumors (*p* > 0.05).

The expression levels of HNF4a in the twenty different malignant tumors were related to different prognoses. It was shown in our study that patients with high HNF4a expression showed a significantly longer overall survival (OS, *p* < 0.05) than patients with low HNF4a expression in BLCA, KIRC, and READ, while patients with high expression of HNF4a exhibited a significantly shorter OS (*p* < 0.05) than patients with low expression of HNF4a in LUSC (Figure 1). There were no significant differences in other malignant tumors (*p* > 0.05). We extracted the mRNA levels of HNF4a from some normal tissues in GTEx and plotted a box diagram of the HNF4a expression. As shown in Supplementary Figure 1, HNF4a mRNA levels increased relatively in kidney, bladder, colorectum, liver, pancreas, and stomach, while relative downregulation is observed in breast, cervix, esophagus, lung, ovary, and uterus.

**Figure 2:**
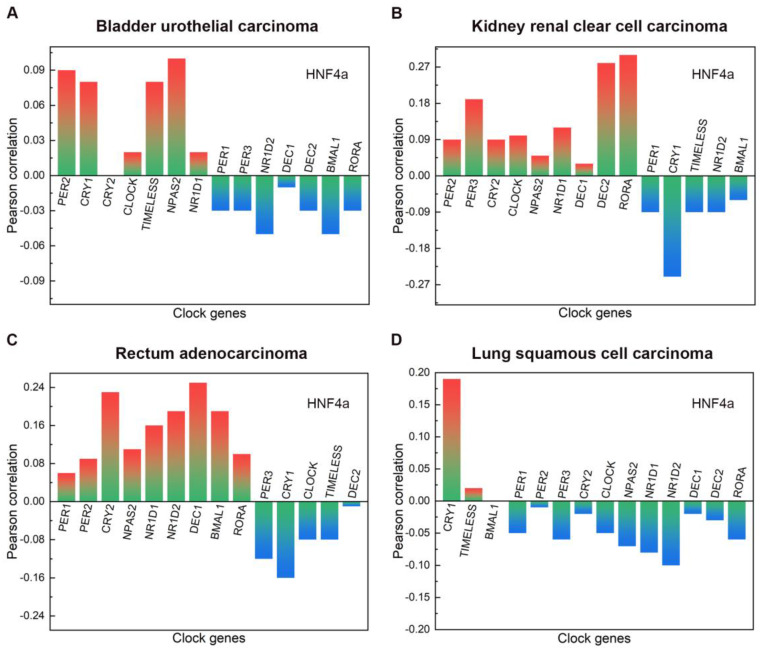
The correlation between HNF4a and other clock genes in BLCA, KIRC, READ, and LUSC. A. In BLCA, HNF4a correlated positively with PER2, CRY1, CLOCK, TIMELESS, NPAS2, and NR1D1 and negatively with PER1, PER3, NR1D2, DEC1-2, BMAL1, and RORA. B. In KIRC, HNF4a correlated positively with PER2-3, CRY2, CLOCK, NPAS2, NR1D1, DEC1-2, and RORA and negatively with PER1, CRY1, TIMELESS, NR1D2, and BMAL1. C. In READ, HNF4a correlated positively with PER1-2, CRY2, NPAS2, NR1D1-2, DEC1, BMAL1, and RORA and negatively with PER3, CRY1, CLOCK, TIMELESS, and DEC2. D. In LUSC, HNF4a correlated positively with CRY1 and TIMELESS and negatively with PER1-3, CRY2, CLOCK, NPAS2, NR1D1-2, DEC1-2, and RORA.

The main clinicopathological features of BLCA are given in Table 1. In BLCA, the high expression rate of HNF4a in the stage group (I+II) was 65.38% (85/130), and that in the stage group (III+IV) was 42.86% (117/273). The high expression rate of HNF4a in the T stage group (T1+T2) was 61.98% (75/121), and that in the T stage group (T3+T4) was 43.43% (109/251). The high expression rate of HNF4a in the lymph node status group (N0) was 53.62% (126/235), and that in the lymph node status group (N1-3) was 39.06% (50/128). Overall, the expression of HNF4a correlated with the stage, T stage, and lymph node status (*p* < 0.05; Table 2) but not with gender, age, metastasis, and radiation (*p* > 0.05). As shown in Figure 2A, HNF4a has a correlation with other clock genes in BLCA, which is reflected in the fact that HNF4a correlates positively with PER2 (P = 0.09), CRY1 (P = 0.08), CLOCK (P = 0.02), TIMELESS (P = 0.08), NPAS2 (P = 0.1), and NR1D1 (P = 0.02) and negatively with PER1 (P = -0.03), PER3 (P = -0.03), NR1D2 (P = -0.05), DEC1 (P = -0.01), DEC2 (P = -0.03), BMAL1 (P = -0.05), and RORA (P = -0.03).

The main clinical and biological features of KIRC are given in Table 3. In KIRC, the high expression rate of HNF4a in the female group was 40.96% (77/188), and that in the male group was 54.78% (189/345). Overall, the expression of HNF4a correlated with gender (*p* < 0.05; Table 4) but not with age, stage, T stage, lymph node status, metastasis, and radiation (*p* > 0.05). As shown in Figure 2B, HNF4a has a correlation with other clock genes in KIRC, which is reflected in the fact that HNF4a correlates positively with PER2 (P = 0.09), PER3 (P = 0.19), CRY2 (P = 0.09), CLOCK (P = 0.1), NPAS2 (P = 0.05), NR1D1 (P = 0.12), DEC1 (P = 0.03), DEC2 (P = 0.28), and RORA (P = 0.3) and negatively with PER1 (P = -0.09), CRY1 (P = -0.25), TIMELESS (P = -0.09), NR1D2 (P = -0.09), and BMAL1 (P = -0.06).

The main clinical and biological features of READ are given in Table 5. The expression of HNF4a was independent of gender, age, stage, T stage, lymph node status, metastasis, and radiation. (*p* > 0.05; Table 6). As shown in Figure 2C, HNF4a has a correlation with other clock genes in READ, which is reflected in the fact that HNF4a correlates positively with PER1 (P = 0.06), PER2 (P = 0.09), CRY2 (P = 0.23), NPAS2 (P = 0.11), NR1D1 (P = 0.16), NR1D2 (P = 0.19), DEC1 (P = 0.25), BMAL1 (P = 0.19), and RORA (P = 0.1) and negatively with PER3 (P = -0.12), CRY1 (P = -0.16), CLOCK (P = -0.08), TIMELESS (P = -0.08), and DEC2 (P = -0.01).

The main clinical and biological features of LUSC are given in Table 7. In LUSC, the low expression rate of HNF4a in the age group (<60 years) was 62.22% (56/90), and that in the age group (≥60 years) was 46.62% (186/399). Overall, the expression of HNF4a correlated with age (*p* < 0.05; Table 8) but not with gender, stage, T stage, lymph node status, metastasis, and radiation (*p* > 0.05). As shown in Figure 2D, HNF4a has a correlation with other clock genes in LUSC, which is reflected in the fact that HNF4a correlates positively with CRY1 (P = 0.19) and TIMELESS (P = 0.02) and negatively with PER1 (P = -0.05), PER2 (P = -0.01), PER3 (P = -0.06), CRY2 (P = -0.02), CLOCK (P = -0.05), NPAS2 (P = -0.07), NR1D1 (P = -0.08), NR1D2 (P = -0.1), DEC1 (P = -0.02), DEC2 (P = -0.03), and RORA (P = -0.06).

## Discussion

The circadian clock exists extensively in various organs and tissues of mammals so that the organism can have a better balance with the rhythm of nature, which has an important biological significance. Mammalian circadian clocks are composed of the master clock and the peripheral clock 29. Under physiological or pathological stimulations, the body can coordinate the output rhythm of the circadian clock driven by suprachiasmatic nucleus and be transmitted finally to the peripheral clock of the peripheral tissues through a series of signal transduction. The peripheral clock can also regulate the rhythmic change of the local circadian clock of the tissues according to the external stimulation and changes to its own microenvironment.

The master clock of mammals consists primarily of two interlocking transcription/translation circuits. The biological clock molecular regulation system in mammals is a negative feedback transcription/translation circuit formed by more than ten biological clock genes, including BMAL1, CLOCK, CRY, PER, Rev-erbs, Rorα, etc. BMAL1 can bind to the CLOCK protein to form a BMAL1/CLOCK heterodimer, then enter the nucleus and bind to the E-box element of the upstream promoter of the PER and CRY genes and activate its transcription. The proteins of PER and CRY in the cytoplasm can be transferred to the nucleus after continuous accumulation, and combined with the D-box elements in the promoter regions of BMAL1 and CLOCK to exert a negative regulatory function and inhibit the transcriptional activity of BMAL1 and CLOCK 30, 31. At the same time, CLOCK and BMAL1 heterodimer can also bind to the E-box element on the promoter of the nuclear receptor gene Rev-erbα/β and RORα/β to promote its gene transcription and start the auxiliary circuit of the clock gene transcription/translation oscillation mechanism. Rev-erbα/β and RORα/β proteins competitively bind to receptor response elements in the promoter region of BMAL1. The RORα/β protein promotes the transcription of BMAL1, while the Rev-erbα/β protein inhibits the transcription of BMAL1, thus producing another level of regulation on the main circuit of circadian rhythm 32-34. The circadian clock regulates the activities of cells, tissues, and organs by regulating many downstream genes to achieve the physiological, biochemical, and complex transformation processes of the body.

HNF4a belongs to the transcription factor of nuclear hormone receptor superfamily 2A, which is mainly found in liver, kidney, and intestinal tissues 35-38. In lung tissue, the content of HNF4a is low and its positive expression can even be used as a useful marker for difficult diagnosis in small biopsy samples 39. The analysis results of HNF4a in the GTEx database also showed consistency. HNF4a was highly expressed in bladder, kidney, and rectum but minimally expressed in the lung under normal circumstances. The expression level or the transcriptional activity of various nuclear receptors (NRs) is related closely to the circadian rhythm. Qu et al. showed that HNF4A can effectively inhibit the transcriptional activity of the CLOCK: BMAL1 heterodimer independently of the indirect action of CRY bridging. Moreover, the authors confirmed that when HNF4a is expressed ectopically, the intrinsic circadian clock can change significantly, indicating the importance of HNF4a in the circadian rhythm of its endogenously expressed cells 40. Caratti's findings revealed that the effect of the glucocorticoid receptor on the energy metabolism and regulation of immunity is felt through the regulation of the HNF4A/HNF6 binding complex by the circadian clock gene NR1D1 in the liver 41. By analyzing the correlation between clock genes, we found that HNF4A correlated negatively with BMAL1 and positively with NR1D1 in BLCA, which was consistent with previous studies.

Bladder cancer is the ninth most commonly diagnosed cancer in the world. About 80% to 90% of bladder cancers are BLCA 42. The pathogenesis of bladder cancer is complicated and related to multiple genes 43, 44. Urine concentration and excretion are regulated by a number of factors, such as vasopressin, thyroxine, and insulin and exhibit distinct circadian rhythms. Circadian clock genes certainly play an important regulatory role in the urinary system, and the malfunctioning of the circadian rhythm could be a contributing mechanism to dysregulation during the development of BLCA. A study by Litlekalsoy et al. analyzed twenty-seven paired tumor and benign tissue samples collected from patients undergoing cystectomy and found that both the oncogene overexpression and reduced transcription of tumor suppressor genes were associated with levels of clock genes 45. In our study, the HNF4a protein expression in BLCA tissues correlated significantly with the tumor pathology (TNM) grade of the clinicopathological data. The higher the T, N stage, the lower the expression level of HNF4a. The malignant degree of the tumor correlated negatively with the expression level of HNF4a, which suggests that HNF4a plays an important negative role in the development of BLCA. We hypothesize that one aspect may be due to declining transcriptional translation levels of the downstream genes associated with cancer caused directly by HNF4a or that HNF4a reverses the abnormal proliferation and differentiation of cells directly. On the other hand, HNF4a may affect the expression levels of other important circadian clock genes, such as elevated BMAL1 levels, which indirectly affect the regulatory genes and cell signaling pathways involved in DNA damage repair, inflammatory response, cellular metabolism, etc., which can enable cancer cells to avoid deterioration and invasion easily; however, the specific molecular mechanism needs to be researched further. There were only 11 cases (5.34%) with distant metastasis in this study. There was no correlation between the M stage and HNF4a expression level, so we speculated that most of the specimens originated from patients undergoing total cystectomy, but patients with distant metastases of the BLCA generally do not undergo total cystectomy. Differences in survival time between patients with different expressions are also seen in the analysis of the overall survival curve. The overall survival time of patients with high expression of the HNF4a protein is long, whereas, the overall survival time of patients with low HNF4a protein expression is short. Therefore, we hypothesize that HNF4A plays a central role in cell-autonomous circadian rhythm oscillation in a tissue-specific manner in BLCA, pointing to its role in the regulation of the circadian clock network in peripheral tissues.

Non-small cell lung cancer (NSCLC) can be classified as nonsquamous carcinoma, including adenocarcinoma (40% of NSCLC cases) and squamous NSCLC (30% of NSCLC cases) 46. With the application of vascular-related factor inhibitors, the prognosis of patients with advanced lung adenocarcinoma has improved significantly. However, the performance of these specific inhibitors in patients with squamous NSCLC is poor. Therefore, understanding and studying the potential molecular targets of squamous NSCLC may open new avenues. It has been reported that HNF4a can regulate the development of human mucinous lung adenocarcinoma by inducing MUC3 47. Currently, there are no studies on HNF4a and LUSC. Our study showed that the higher the level of HNF4a expression in LUSC, the worse the patient's prognosis, which is significantly different from the results in BLCA, KIRC, and READ. However, the correlation between HNF4a and clinical pathology is not obvious. The specific mechanism of action of HNF4a in LUSC is worthy of our subsequent research and discussion.

The evaluation of the expression of a single clock gene is limited. The studies on transcription data between the different clock genes have shed light on the question of whether the clock-work acts as a system and not only by single gene aberrations. In different tumors, the mutual influence of different clock genes leads to different outcomes, which reminds us that when we conduct basic research on a single clock gene, we should pay attention to the promotion or inhibition influence of other clock genes. It is the regulation of these systems that makes the circadian rhythm so orderly, and even forms characteristic asynchronous circadian rhythms in cancers.

In conclusion, this study shows that HNF4a could be a tumor suppressor gene, and its loss of expression in BLCA tissues is likely to promote the occurrence and progression of BLCA. The high expression of HNF4a in the bladder mucosa of patients may help to inhibit the occurrence and development of tumors and could have a certain guiding role in the early diagnosis and treatment of patients. The HNF4a protein could be used as one of the prognostic indicators for patients with BLCA, and it could become a possible alternative target for BLCA treatment. The combined detection of the HNF4a protein and other factors could improve the prediction of the overall survival of patients with BLCA greatly and provide a basis for early detection and early clinical treatment intervention. Therefore, we intend to explore the related mechanism of the HNF4a gene's low-expression in BLCA further and hope to provide a clinical basis for the early diagnosis and prognosis of bladder cancer patients.

## Supplementary Material

Supplementary figure S1.

## Figures and Tables

**Table 1:** The clinical characteristics of bladder urothelial carcinoma.

Variables	Case, n (%)
**Age (years)**	
<60	87 (21.48%)
≥60	317 (78.27%)
NA	1 (0.25%)
**Stage**	
I + II	130 (32.10%)
III + IV	273 (67.41%)
NA	2 (0.49%)
**T stage**	
T1 + T2	121 (29.87%)
T3 + T4	251 (61.98%)
TX	1 (0.25%)
NA	32 (7.90%)
**Lymph node status**	
N0	235 (58.02%)
N1-3	128 (31.61%)
NX	36 (8.89%)
NA	6 (1.48%)
**Metastasis**	
M0	195 (48.15%)
M1	11 (2.72%)
MX	196 (48.39%)
NA	3 (0.74%)
**Gender**	
Female	106 (14.67%)
Male	299 (85.33%)
**Radiation**	
No	359 (67.39%)
Yes	20 (22.83%)
NA	26 (9.78%)

NA, not available.

**Table 2:** The clinicopathological correlation of HNF4a expression in bladder urothelial carcinoma.

Variables	HNF4a		χ^2^	*p*-values^ a^
	Low expression	High expression		
**Gender**			1.761	0.185
Female	59	47		
Male	144	155		
**Age (years)**			2.476	0.116
<60	37	50		
≥60	165	152		
**Stage**			17.877	0.000*
I + II	45	85		
III + IV	156	117		
**T stage**			11.247	0.001*
T1 + T2	46	75		
T3 + T4	142	109		
**Lymph node status**			7.028	0.008*
N0	109	126		
N1-3	78	50		
**Metastasis**			1.604	0.205
M0	86	109		
M1	7	4		
**Radiation**			3.246	0.072
No	182	177		
Yes	6	14		

^a^ Pearson Chi-square. * Indicates *p* < 0.05.

**Table 3:** The clinical characteristics of kidney renal clear cell carcinoma.

Variables	Case, n (%)
**Age (years)**	
<60	245 (45.97%)
≥60	287 (53.85%)
NA	1 (0.18%)
**Stage**	
I + II	324 (60.79%)
III + IV	207 (38.84%)
NA	2 (0.37%)
**T stage**	
T1 + T2	342 (64.17%)
T3 + T4	191 (35.83%)
**Lymph node status**	
N0	240 (45.03%)
N1-3	16 (3.00%)
NX	277 (51.97%)
**Metastasis**	
M0	422 (79.17%)
M1	79 (14.82%)
MX	30 (5.63%)
NA	2 (0.38%)
**Gender**	
Female	188 (14.67%)
Male	345 (85.33%)
**Radiation**	
No	141 (26.45%)
Yes	2 (0.38%)
NA	390 (73.17%)

NA, not available.

**Table 4:** The clinicopathological correlation of HNF4a expression in kidney renal clear cell carcinoma.

Variables	HNF4a		χ^2^	*p*-values^ a^
	Low expression	High expression		
**Gender**			9.304	0.002*
Female	111	77		
Male	156	189		
**Age (years)**			0.475	0.491
<60	119	126		
≥60	148	139		
**Stage**			2.184	0.139
I + II	154	170		
III + IV	112	95		
**T stage**			1.304	0.253
T1 + T2	165	177		
T3 + T4	102	89		
**Lymph node status**			0.051	0.821
N0	127	113		
N1-3	8	8		
**Metastasis**			1.534	0.215
M0	203	219		
M1	44	35		
**Radiation**			1.788	0.181
No	74	67		
Yes	2	0		

^a^ Pearson Chi-square. * Indicates *p* < 0.05.

**Table 5:** The clinical characteristics of rectum adenocarcinoma.

Variables	Case, n (%)
**Age (years)**	
<60	41 (42.27%)
≥60	56 (57.73%)
**Stage**	
I + II	41 (42.27%)
III + IV	47 (48.45%)
NA	9 (9.28%)
**T stage**	
T1 + T2	18 (18.56%)
T3 + T4	77 (79.38%)
NA	2 (2.06%)
**Lymph node status**	
N0	43 (44.33%)
N1-3	50 (51.55%)
NX	2 (2.06%)
NA	2 (2.06%)
**Metastasis**	
M0	67 (69.07%)
M1	13 (13.41%)
MX	14 (14.43%)
NA	3 (3.09%)
**Gender**	
Female	44 (45.36%)
Male	53 (54.64%)
**Radiation**	
No	73 (75.26%)
Yes	11 (11.34%)
NA	13 (13.40%)

NA, not available.

**Table 6:** The clinicopathological correlation of HNF4a expression in rectum adenocarcinoma.

Variables	HNF4a		χ^2^	*p*-values^ a^
	Low expression	High expression		
**Gender**			0.099	0.752
Female	23	21		
Male	26	27		
**Age (years)**			0.495	0.482
<60	19	22		
≥60	30	26		
**Stage**			1.142	0.285
I + II	18	23		
III + IV	26	21		
**T stage**			0.329	0.566
T1 + T2	8	10		
T3 + T4	40	37		
**Lymph node status**			1.766	0.184
N0	19	24		
N1-3	29	21		
**Metastasis**			0.268	0.605
M0	36	31		
M1	8	5		
**Radiation**			0.785	0.376
No	37	36		
Yes	4	7		

^a^ Pearson Chi-square. * Indicates *p* < 0.05.

**Table 7:** The clinical characteristics of lung squamous cell carcinoma.

Variables	Case, n (%)
**Age (years)**	
<60	90 (18.18%)
≥60	399 (80.61%)
NA	6 (1.21%)
**Stage**	
I + II	90 (18.18%)
III + IV	401 (81.01%)
NA	4 (0.81%)
**T stage**	
T1 + T2	402 (81.21%)
T3 + T4	93 (18.79%)
**Lymph node status**	
N0	316 (58.02%)
N1-3	173 (31.61%)
NX	6 (8.89%)
**Metastasis**	
M0	407 (82.22%)
M1	7 (1.41%)
MX	77 (15.56%)
NA	4 (0.81%)
**Gender**	
Female	129 (26.06%)
Male	366 (73.94%)
**Radiation**	
No	378 (76.36%)
Yes	53 (10.71%)
NA	64 (12.93%)

NA, not available.

**Table 8:** The clinicopathological correlation of HNF4a expression in lung squamous cell carcinoma.

Variables	HNF4a		χ^2^	*p*-values^ a^
	Low expression	High expression		
**Gender**			0.007	0.935
Female	67	67		
Male	179	182		
**Age (years)**			7.154	0.007*
<60	56	34		
≥60	186	213		
**Stage**			0.198	0.656
I + II	202	199		
III + IV	43	47		
**T stage**			0.168	0.682
T1 + T2	198	204		
T3 + T4	48	45		
**Lymph node status**			0.093	0.760
N0	158	158		
N1-3	84	89		
**Metastasis**			0.077	0.781
M0	211	196		
M1	4	3		
**Radiation**			0.739	0.390
No	195	183		
Yes	24	29		

^a^ Pearson Chi-square. * Indicates *p* < 0.05.
