# Supplementary material for: Research on the circadian clock gene HNF4a in different malignant tumors
Source: Int J Med Sci. 2021 Jan 21;18(6):1339–47. doi: 10.7150/ijms.49997 (PMC7893568; doi:10.7150/ijms.49997)

Supplementary Figure

Research on the circadian clock gene HNF4a in different malignant tumors

Meng-jun Qiu, Li Zhang, Xie-fan Fang, Qiu-ting Li, Li-sheng Zhu, Bin Zhang, Sheng-li Yang, Zhi-fan Xiong

Supplementary Figure 1. Expression profile for HNF4a in some human normal tissues.

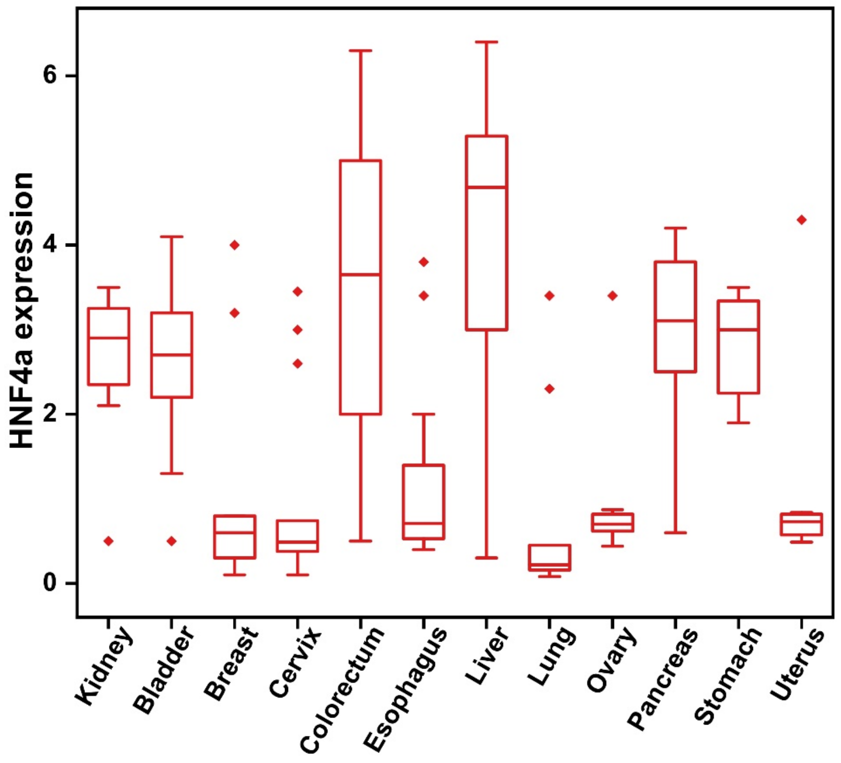

Supplement: Supplementary file 1 — Supplementary figure S1. [file ijmsv18p1339s1.pdf]
